# Treatment of Nævus

**Published:** 1848-07

**Authors:** 


*Treatment of Nævus.*—Dr. Cordier, in a paper addressed to the Academy of Sciences, states, that in order to destroy or modify nævi materni, he introduces white lead under the skin by means of needles. This operation is soon followed by a little inflammation, phlyctenæ, and slight eschars. When the latter fall, the coloring matter employed, (the white lead,) which is very analogous to the colour of the parts around, takes the place of the nævus, and remains indelibly. (We suspect that this is rather far-fetched, and would hardly bear the test of practice.)—*Ibid.*

